# Interaction between granulin A and enolase 1 attenuates the migration and invasion of human hepatoma cells

**DOI:** 10.18632/oncotarget.16328

**Published:** 2017-03-17

**Authors:** Xiaoliang Chen, Huanli Xu, Ning Wu, Xiujun Liu, Gan Qiao, Shuonan Su, Ye Tian, Ru Yuan, Cong Li, Xiaohui Liu, Xiukun Lin

**Affiliations:** ^1^ Department of Pharmacology, Capital Medical University, Beijing 100069, China; ^2^ Institute of Oceanology, Chinese Academy of Science, Qingdao 266003, China; ^3^ Institute of Medicinal Biotechnology, Chinese Academy of Medical Science, Beijing 100050, China

**Keywords:** enolase 1, granulin A, invasion, migration, protein-protein interaction

## Abstract

Granulin A (GRN A), a peptide with a molecular 6 *kDa*, is derived from proteolysis of progranulin (PGRN). Previous study in our laboratory has shown that GRN A is able to inhibit cancer cell growth significantly. In the present study, we confirmed that GRN A can bind to α-enolase (ENO1) specifically as analyzed using Pull-down/MS approaches. The interaction of GRN A with ENO1 was further confirmed by Western blotting and Surface plasmon resonance (SPR) analysis. Treatment of human HepG-2 cells with GRN A inhibited cancer cell growth as well as migration and invasion of cancer cells as analyzed by the 3-(4,5-dimethylthiazol-2-yl)-2,5-diphenyl tetrazoliumbromide (MTT) and Scratch wound healing assay as well as Transwell experiments. Additionally, GRN A treatment results in augmentation of glucose uptake in cancer cells. Further study reveals that higher expression of ENO1 reversed the inhibitory effects of GRN A on migration and invasion of HepG-2 cells. The increase of glucose uptake, as well as the expression of apoptosis-related genes, is also reversed in cells overexpressing ENO1. The study provides solid evidence that there is the interaction between GRN A and ENO1 and the interaction is responsible for the effects of GRN A on glucose uptake as well as cancer cell migration and invasion.

## INTRODUCTION

Granulins (GRNs), also known as epithelins, are a family of about 6 *kDa* peptides that derived from proteolysis of progranulin (PGRN). The GRNs family including GRN A, B, C, D, E, F, G, contain 12 cysteine residues with diverse functions [1–3]. It is well established GRNs play an important role in mammalian cell growth, acting as agonistic and antagonistic in cell development [4]. GRN A is initially found and purified from human leukocytes and rat bone marrow [5], and the peptide was confirmed to display proliferation inhibition on human epidermoid carcinoma A431 cells and breast cancer MD-MBA-468 cells [6, 7]. Previous studies in our laboratory also revealed that GRN A induced cancer cell apoptosis in several human cancer cells [8]. However, the exact targets of the polypeptide are unknown and the underlying mechanism needed to be addressed.

Metastasis and invasion play critical roles in tumor malignancy and antimetastasis represents an important strategy on the treatment of cancer. Enolases, catalyzing the conversion of 2-phosphoglycerate (2-PG) to phosphoenolpyruvate (PK), besides its role in glycolysis, also play role in cancer metastasis. There are three different isoforms enolase; α-enolase (ENO1), γ-enolase (ENO2), and β-enolase (ENO3). ENO1 with a molecular weight of 48 *kDa* is expressed in both the cytoplasm as well as cell membrane [9]. ENO1 is able to promote cell growth via FAK/PI3K/AKT pathway [10]. Recent study also shows that ENO1 activates pericellular plasminogen, resulting in accelerating degradation of the extracellular matrix and elevation of invasion and metastasis of tumor cells [9, 11]. However, the regulation of ENO1 in cancer cells is not clear. In addition, ENO1 is usually over-expressed in tumor cells. Knocking down the expression of ENO1 results in suppression of cell growth, clone formation, and inhibition of the migration and invasion of cancer cells [11, 12]. The enzyme is considered to be a promising target for the treatment of tumor.

In the present study, the targeted protein of GRN A was identified using pull-down/SDS-PAGE/LC-MS analysis. The interaction between GRN A and ENO1 was investigated using Western blotting and SPR analysis. The effect of GRN A on migration and invasion of cancer cells was studied using the Scratch wound healing assay and the Transwell assays. The underlying mechanism was further illustrated by checking the effect of GRN A on the expression of related proteins using Western blotting assay.

## RESULTS

### GRN A inhibited the growth and induced cells apoptosis

**Figure 1:**
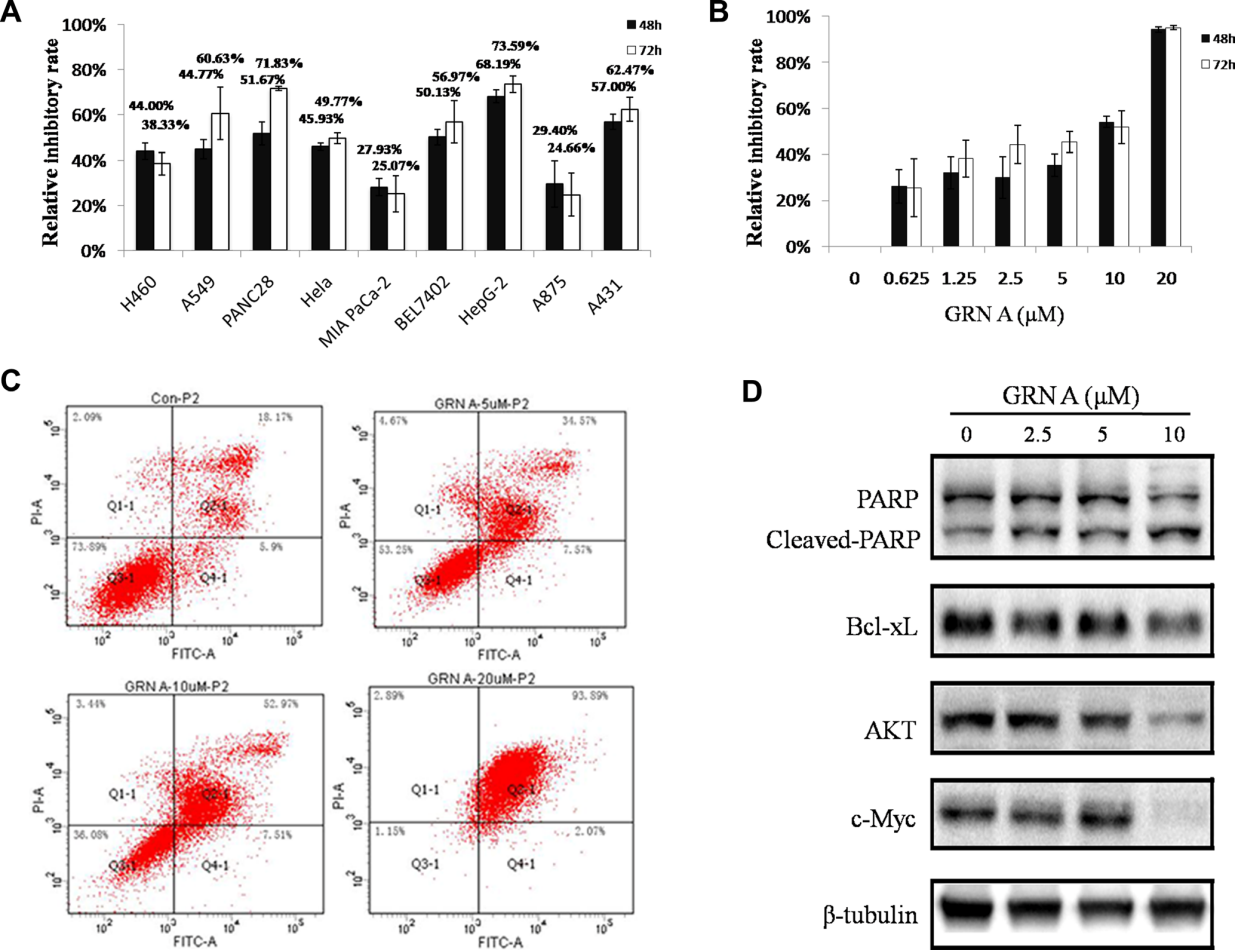
GRN A inhibited the growth and induced apoptosis in cancer cells MTT assay was performed to determine the effect of GRN A on cell growth as described in Materials and Method section. The effect of GRN A on the growth of different cells was presented in (**A**), while (**B**) indicated a dose-dependent assay of GRN A on HepG-2 cells. (**C**) represented the GRN A on cell apoptosis as analyzed using flow cytometry. The expression of apoptosis related-proteins were shown in (**D**) as analyzed using Western blotting.

MTT assay was performed to evaluate the anti-proliferative effects of GRN A against several cell lines. The results revealed that GRN A possessed a significant growth-inhibition effect on cancer cell lines (Figure 1A). After treated with GRN A (10 μM) in serum-free DMEM media for 72 h, the relative inhibitory rate on PANC28, HepG-2, A431 were 71.83 ± 0.96, 73.59 ± 3.64, 62.47 ± 13.46% respectively. Among these cell lines, HepG-2 cells were much more sensitive than that of the other cells lines with an IC50 value of 5.76 μM (Figure 1B). In our next experiments, HepG-2 cells were selected for further study.

To further confirm GRN A induced apoptotic activity, flow cytometry analysis was performed using V-FITC /PI double-staining assay. The results revealed that a dose-dependent increase of total apoptotic cells was observed in cells treated with GRN A; the percentage of total apoptotic cells was 24.07% in untreated cells, whereas the percentages of total apoptotic cells were 42.14, 60.48, 95.96% in the HepG-2 cells treated with 5, 10 and 20 μM GRN A, respectively (Figure 1C). The percentages of late apoptotic cells induced by GRN A at the concentrations of 5, 10 and 20 μM were 34.57, 52.97 and 93.89%, respectively. These results suggest that GRN A induces cell death via apoptotic pathway.

Western blotting analysis was performed to investigate the underlying mechanism regarding the GRN A induced cell apoptosis. The results showed that the expression of anti-apoptosis proteins, including Bcl-xL, AKT, c-Myc, were decreased in a dose-dependent manner in cells treated with GRNA. Meanwhile, the expression of PARP was also diminished, but the expression of cleaved-PARP was increased (Figure 1D).

### Distribution of GRN A in HepG-2 cells

**Figure 2:**
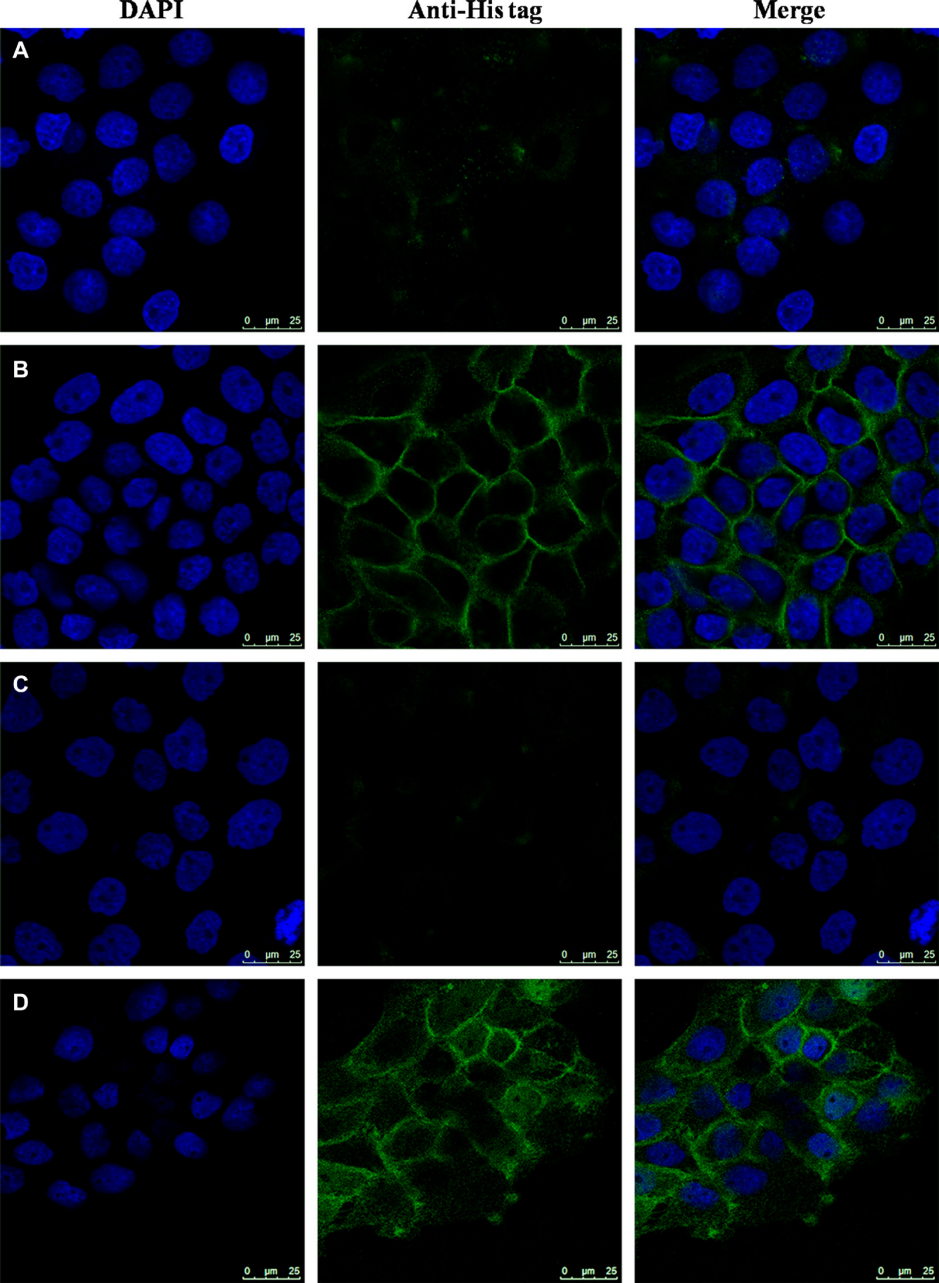
Distribution of GRN A in HepG-2 cells The cells were cultured at 37°C for 24 h and treated without (**A**, **C**) or with (**B**, **D**) GRN A (5 μM). After incubation for another 24 h, cells were fixed with 4% paraformaldehyde and permeabilized with (C, D) or without (A, B) 0.1% triton X-100. The cells were further incubated with a mouse anti-His antibody overnight at 4°C. Subsequently, the cells were incubated with fluorescein isothiocyanate (FITC)-conjugated goat anti-mouse., and the cell image was observed using a Leica TCS SP8 confocal system (Leica microsystems, Germany).

The localization of GRN A was analyzed using Confocal imaging experiment. HepG-2 cells were treated without or with GRN A for 24 h. The results showed that GRN A mainly located in the cell membrane in non-penetrated analysis (Figure 2B). However, GRN A was also observed in both cell membrane and cytoplasm when treated with 0.1% triton X-100 (Figure 2D). These results suggested that GRN A was distributed in both the cell membrane and cytoplasm.

### ENO1 was identified as the molecular target interacted with the GRN A

**Figure 3:**
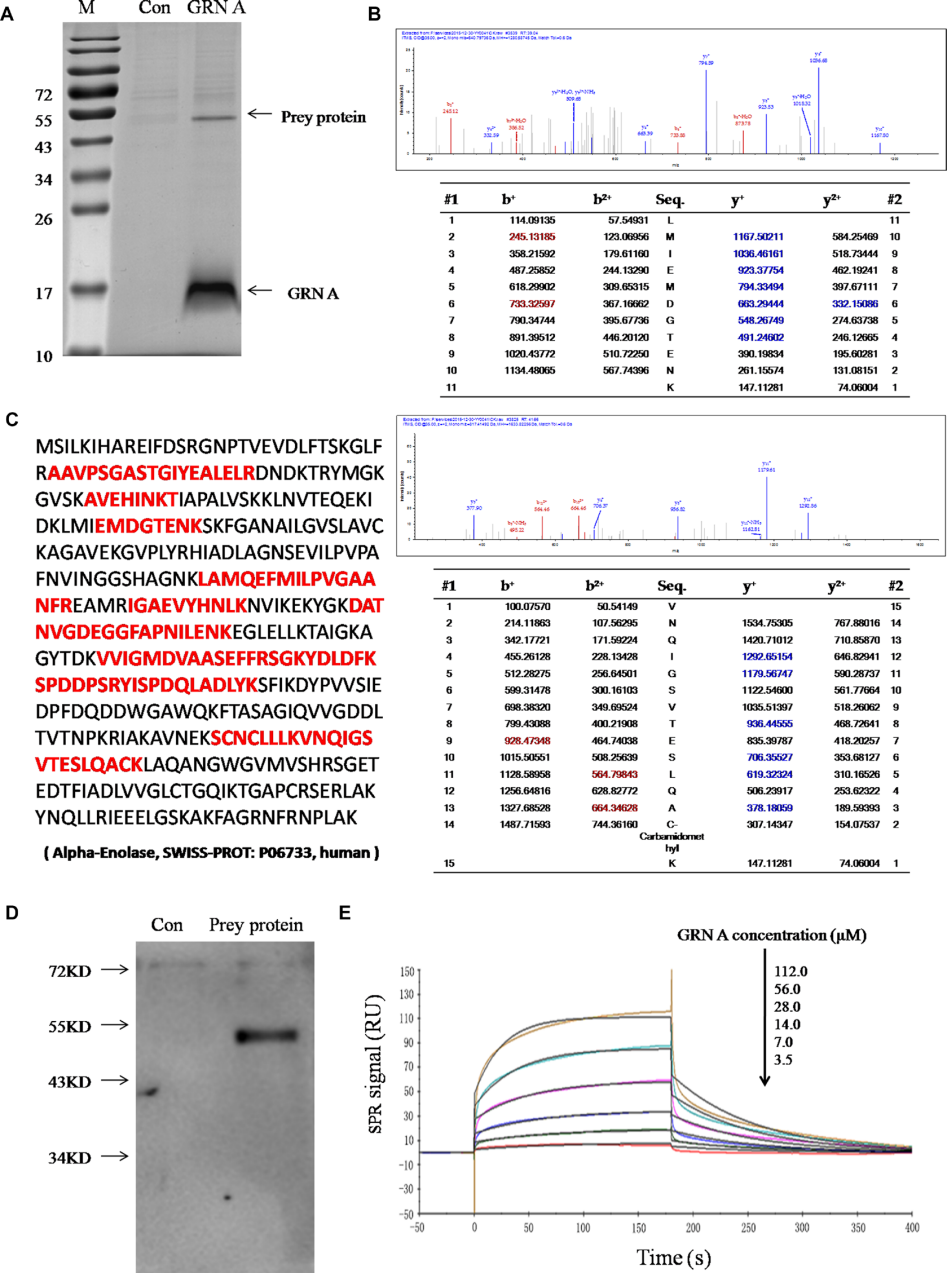
Interaction of GRN A with ENO1 (**A**) SDS–PAGE/His-pull down assay. SDS–PAGE/His-pull down assay was performed as described in the Materials and Method section. The prey protein band was indicated by an arrow. (**B**)Spectra of the prey peptide as analyzed by LC-MS/MS, which were identified as ENO1. (**C**) Amino acid sequence of ENO1. The red font represented the sequence analyzed by MS. (**D**) Western blotting analysis. The gel was immunoblotted using anti-ENO1 monoclonal antibody. (**E**) SPR analysis. The interaction of GRN A and ENO1 was analyzed using BIACORE T200.

To begin identification of the molecular target of GRN A, we initially use Pull-down assay to identify the cellular target protein. Cells treated with or without GRN A were lysed and the lysates were incubated with His-resin. The absorbed proteins on the resins were eluted, and SDS-PAGE analysis was performed to check the preyed proteins by the His-resins. As shown in Figure 3A, the preyed protein appeared obviously at the band with a molecular weight of about 50 *kDa* and the protein was identified as ENO1 using LC-MS/MS analysis (Figure 3B, 3C).

To further confirm the interaction between GRN A and ENO1, Western blotting analysis was carried out. As shown in Figure 3D, the monoclonal His-antibody is able to bind toENO1 specifically. The interaction of GRN A and ENO1 was further investigated using SPR analysis. The results showed that the equilibrium dissociation constant KD value of ENO1 and GRN A was 56.04 μM (K_off_ = 0.01317 Ms^−1^, K_on_ = 235.1s^−1^) (Figure 3E). These results further confirmed the interaction between ENO1 and GRN A.

### GRN A induced cellular glucose uptake

**Figure 4:**
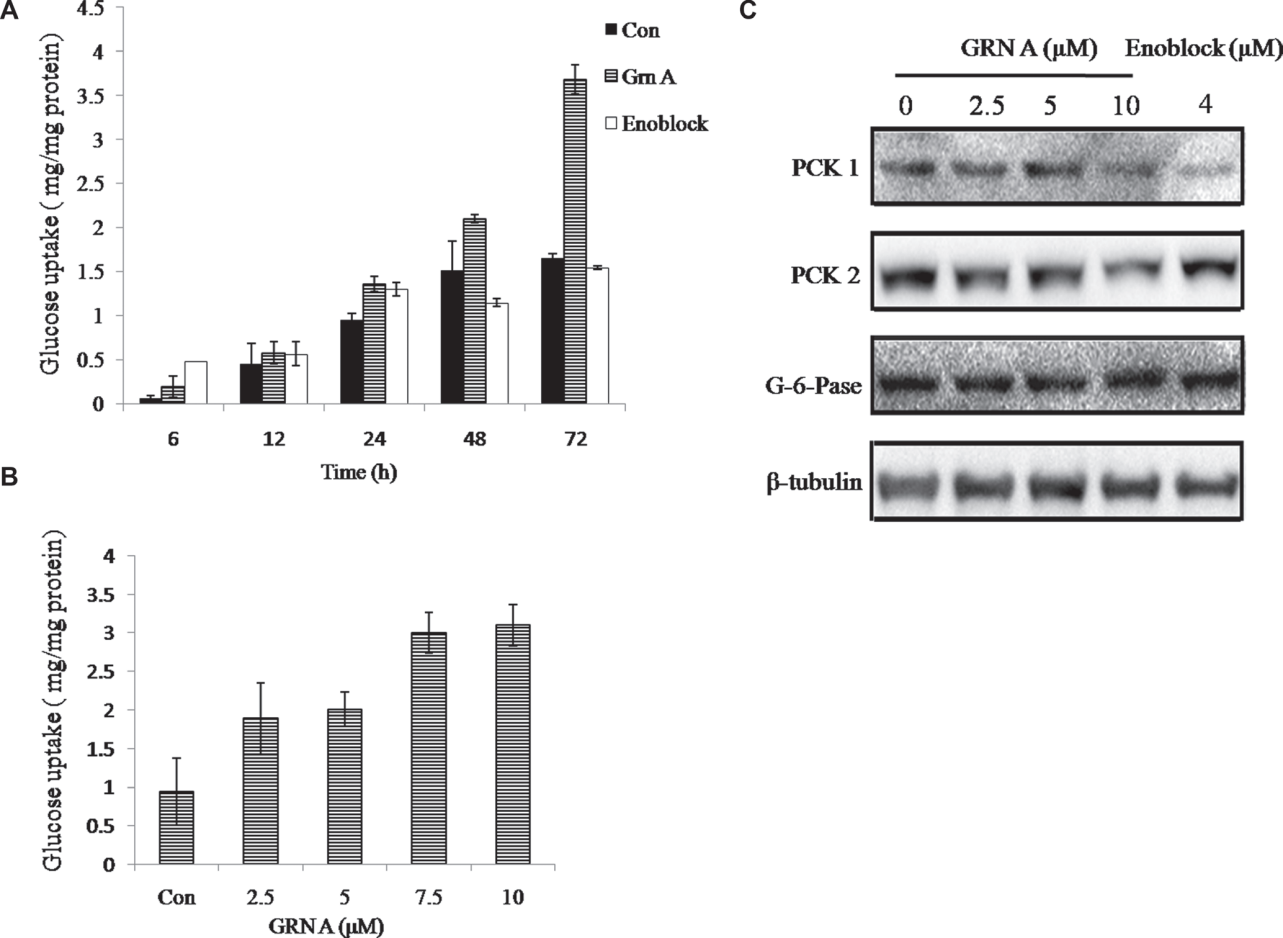
GRN A induced glucose uptake Cell (1 × 10^5^) was seeded in a 60 mm culture dish at 37°C for 24 h, and then the cells were treated with or without GRNA (5 μM) in 1% serum DMEM. The protein concentration and the levels of glucose uptake of the culture media were analyzed as described in Materials and Method section. (**A**) Effect of GRN A on cellular glucose uptake in HepG-2 cells. (**B**) Dose-dependent effect of GRN A on cellular glucose uptake. (**C**) Western blotting analysis. Hepg-2 (2 ×10^5^) was seeded in 60 mm culture dish at 37°C for 24 h, and then the cells were treated with GRNA or enoblock for 48 h. Related protein expression was determined by Western blotting analysis as described in Materials and Method section.

Our results have confirmed that GRN A is able to interact with ENO1 specifically. In our next investigation, we try to dissect the function of the interaction between GRN A and ENO1. Recent study by Jung DW et al identified an ENO1 inhibitor, AP-III-a4 (Cas no. 1177827-73-4), called enoblock, which can bind with ENO1 [13]. Paralleled experiments were performed to compare the effect of GRN A and enoblock on glucose uptake. The results showed that treatment of the HepG-2 cells with GRN A increased the glucose uptake significantly, similar with that of the cells treated with enoblock (Figure 4A, 4B). These results suggested that GRN A is able to induce glucose uptake, and served as an ENO1 inhibitor. To confirm the mechanism by which GRN A promotes glucose uptake, the expression of some key enzymes related to gluconeogenesis were tested. As shown in Figure 4C, the expression of phosphoenolpyruvate carboxykinase 1 (PCK1), and phosphoenolpyruvate carboxykinase 2 (PCK2) were decreased. However, there is no significant change on the expression of glucose-6-phosphatase (G-6-pase). These results suggested that GRN A can increase the gluconeogenesis via inhibiting PCK1 and PCK2.

### GRN A inhibited cells migration and invasion

**Figure 5:**
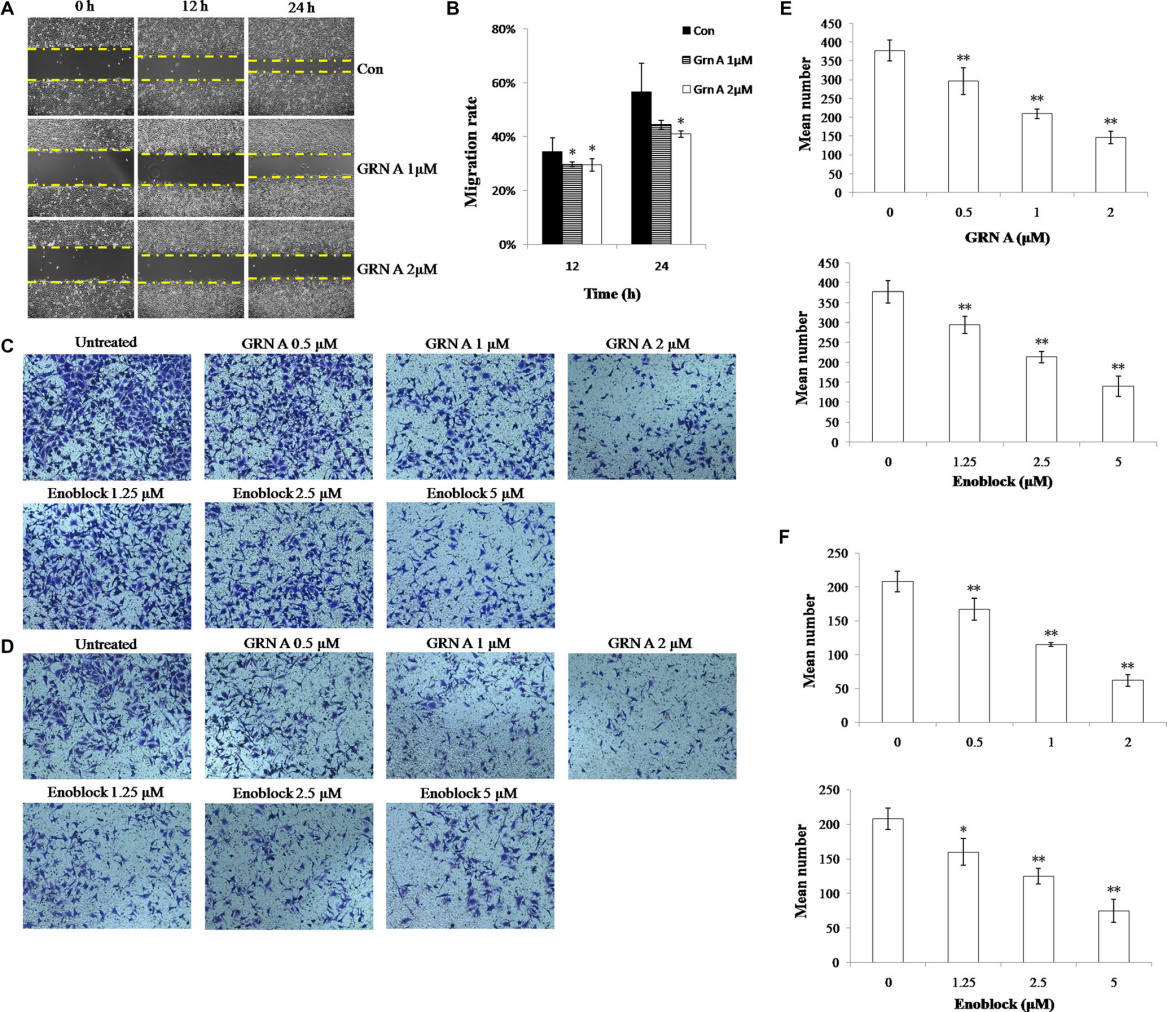
GRN A inhibited cells migration and invasion in HepG-2 cells The effect of GRN A on cell migration and invasion was analyzed as described in Materials and Method section. Scratch wound healing assay was performed to determine the effect of GRN A on cell migration (**A**), while (**B**) is the quantitative result of Figure 5A. (**C**) and (**D**) represented the effect of GRN A on cell migration and invasion as determined using Transwell assay, while (**E**) and (**F**) indicated the quantitative result of Figure 5C, and 5D. The effect of enoblock on cell invasion was performed as a positive control. *n* > 3 experimental repeats, *p < 0.05, **p < 0.01versus control was considered significant.

Previous study has shown that ENO1 is related to cell invasion and migration via interacting with urokinase-type plasminogen activator (uPA), urokinase-type plasminogen activator receptor (uPAR), and plasminogen [9]. To determine if treatment of cancer cells with GRN A affected cell motility, the Scratch wound healing assay was performed. As shown in Figure 5A, compared with the control group, the migration was remarkably inhibited in cells treated with GRN A (1 or 2 μM). Transwell assay also indicated that treatment of the cancer cells with GRN A inhibited cell mobility (Figure 5B); the number of cells migrated to the opposite side were 378 ± 28, 297 ± 35, 210 ± 13, 146 ± 17, when treated the cells with GRN A at a concentration of 0, 0.5, 1.0, 2.0 μM respectively. Treatment of the cancer cells with enoblock (from 0 to 5 μM) also displayed similar results; cells migrated to the opposite side were 378 ± 28, 295 ± 22, 214 ± 14, 141 ± 26, when treated with enoblock at a concentration of 0, 1.25, 2.50, 5.00 μM, respectively. Moreover, GRN A treatment also inhibited cancer cell invasion (Figure 5C) in a dose-dependent manner, and the number of cells invaded to the opposite side were 208 ± 16, 167 ± 16, 115 ± 3, 62 ± 9, when treated the cells with GRN A at a concentration of 0, 0.5, 1.0, 2.0 μM respectively. Similar results were also found when treating the cells with enoblock. These results confirmed that GRN A is able to interfere with the metastasis and inhibits migration and invasion of cancer cells.

### Overexpression of ENO1 promoted cell migration and invasion and reversed the effects of GRN A in HepG-2 cells

**Figure 6:**
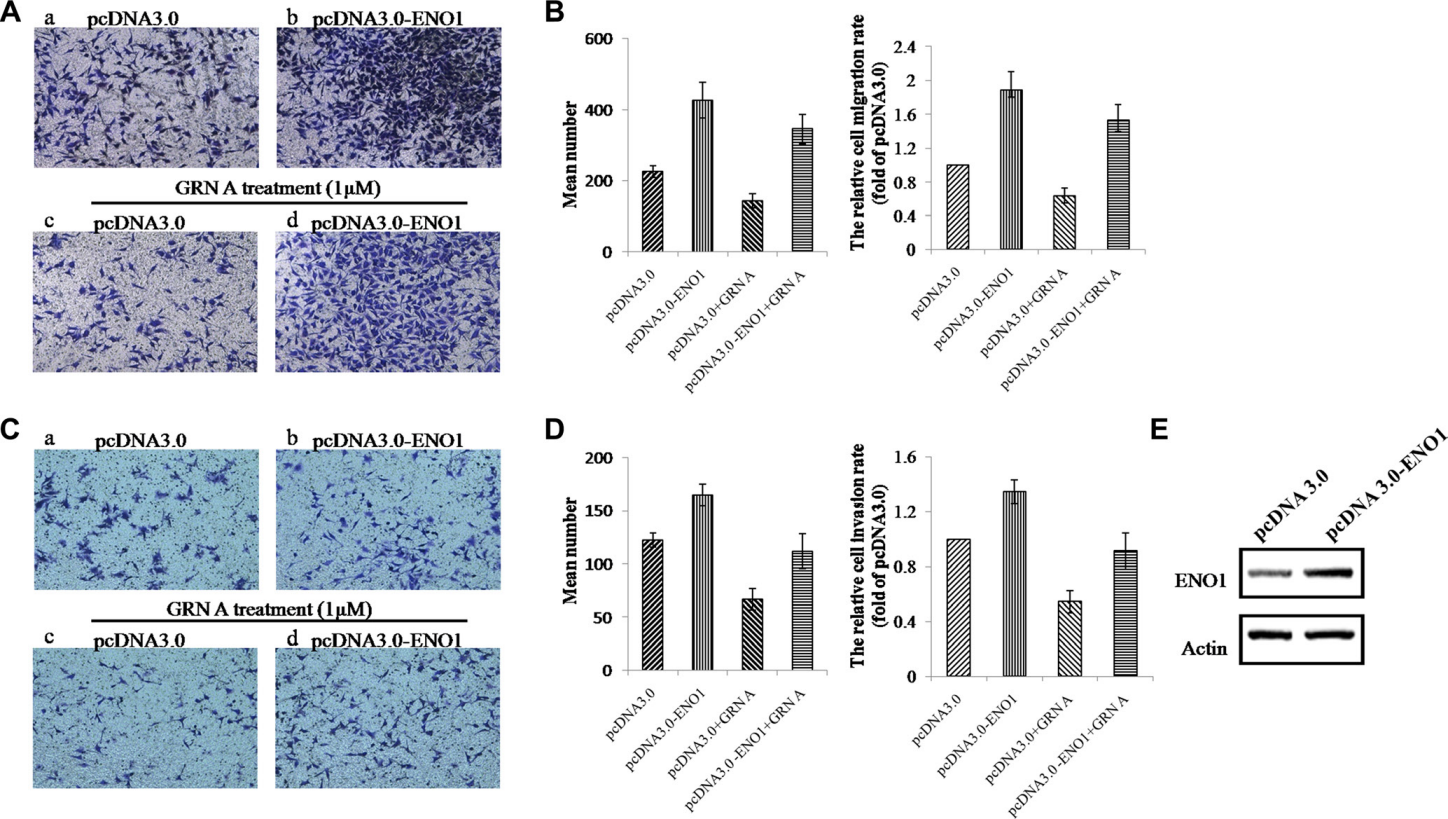
Overexpression of ENO1 reversed the effect of GRN A on cell migration and invasion (**A**) and (**B**) indicated the migration and invasion in ENO1 overexpressing cells as analyzed using Transwell experiment, while (**C**), and (**D**) represented the quantitative results of (A) and (B). (**E**) indicated the levels of ENO1 in transfected with the ENO1 construct.

To study if the inhibition of migration and invasion of GRN A is related to the targeted protein ENO1, we studied the effect of GRN A on cells over-expressing ENO1. As shown in Figure 6A, the migration cells number in HepG-2 cells transfected with ENO1 increased from 227 ± 15 to 427 ± 49 (Figure 6Aa, 6Ab), suggesting that ENO1 is capable of enhancing the ability of cell migration. Additionally, treatment with GRN A inhibited the cell migration significantly; the migration cells number decreased from 227 ± 15 to 144 ± 21 in cells transfected with control constructs (Figure 6Aa, 6Ac)). In contrast, overexpression of ENO1 reversed the effect of GRN A on migration of cancer cells; the number of migrated cells was increased to 346 ± 46 in ENO1 transfected cancer cells treated with GRN A, compared with that (144 ± 21) in cells transfected with the control plasmid (Figure 6Ac, 6Ad). Similarly, ENO1 overexpression attenuated the GRN A associated inhibitory effect of cell invasion. As shown in Figure 6B, the invaded cells number transfected with ENO1 increased from 123 ± 7 to 165 ± 10, indicating that ENO1 is able to promote the ability of cell invasion (Figure 6Ba, 6Bb)). In addition, treatment with GRN A inhibited the cell invasion significantly; the invasion cells number decreased from 123 ± 7 to 67 ± 10 (Figure 6Ba, 6Bc). However, overexpression of ENO1 reversed the effect of GRN A on migration of cancer cells significantly; the number of migrated cells was increased to 112 ± 16 in ENO1 transfected cancer cells treated with GRN A, compared with that in cells transfected with the control constructs (Figure 6Bc, 6Bd). These results suggested that the effect of GRN A on cell migration and invasion is associated with ENO1; overexpression of ENO1 promotes the cell migration and invasion.

### Overexpression of ENO1 enhanced the expression of apoptosis and glucose metabolic-related proteins

**Figure 7:**
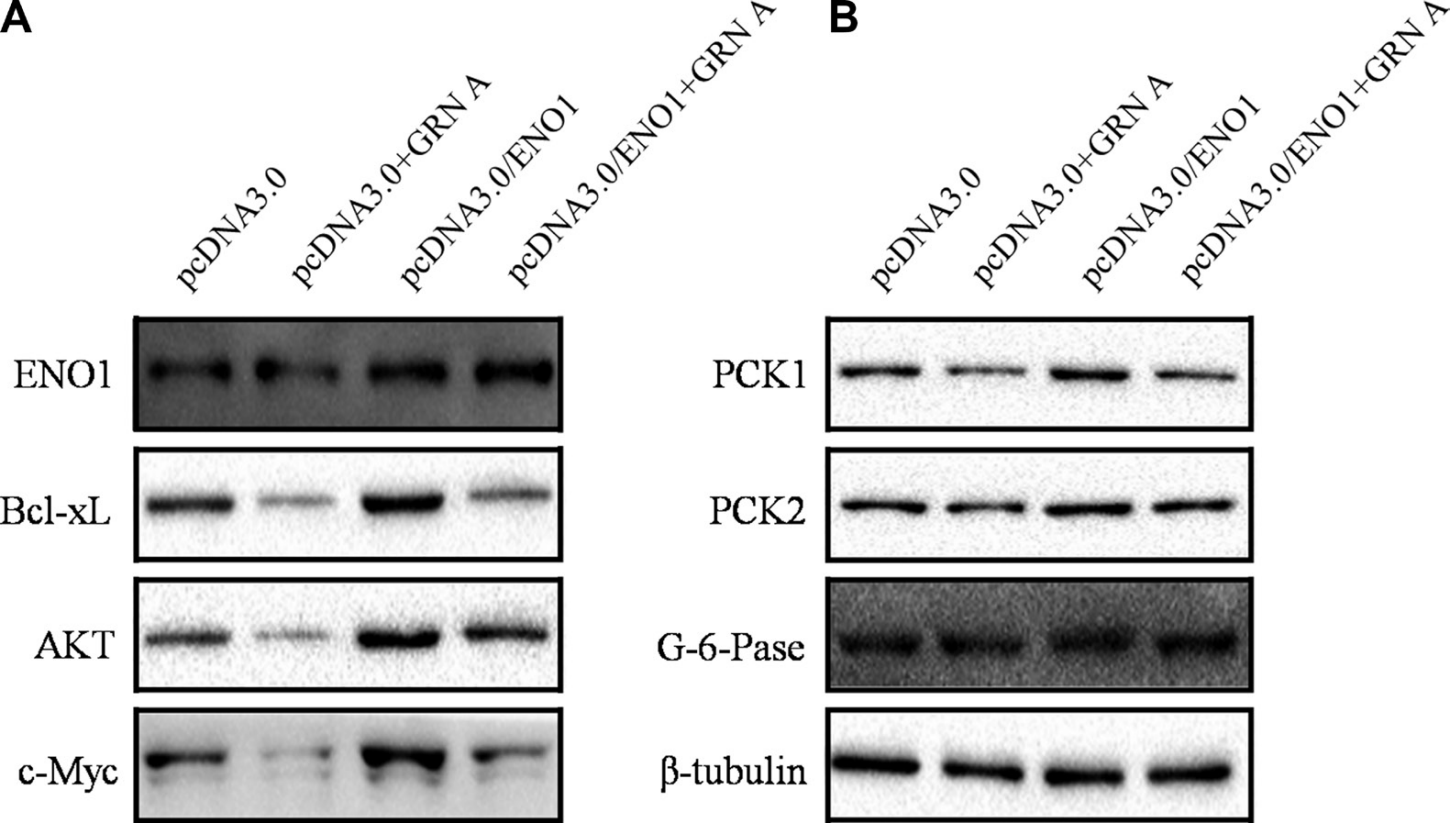
Overexpression of ENO1 enhanced the expression of apoptosis and glucose metabolic-related proteins The expression of apoptosis proteins in cells overexpressing ENO1 was determined using Western blotting as described in Materials and Method section (**A**). The expression of gluconeogenesis-related protein in cells overexpressing ENO1 was presented in (**B**).

We then studied if over-expression of ENO1 affects the expression of apoptosis proteins using Western blotting analysis. The results showed that the expression of anti-apoptosis proteins, displayed opposite effects between normal HepG-2 cells and the cells over-expression of ENO1 when treated with GRN A; the expression of Bcl-xL and c-Myc are diminished significantly in cells treated with GRN A (Figure 1D). However, the expression of these genes was elevated significantly in cells over-expressing ENO1 (Figure 7A). The results indicated that GRN A induced cell apoptosis is associated with ENO1. Similar results were also found in gluconeogenesis proteins; GRN A treatment results in inhibition of the expression of PCK1 and PCK2 significantly (Figure 4C). However, these genes expression was enhanced obviously in cells over-expressing ENO1 (Figure 7B); suggesting that the effect of GRN A on glucose uptake is also associated with ENO1.

## DISCUSSION

In the present report, we provide solid evidence that there is interaction between GRN A and ENO1; GRN A serves as an inhibitor of ENO1. Previous study has shown that an ENO1 inhibitor AP-III-a4, called enoblock, is able to bind with ENO1 and increase the glucose uptake significantly. Our present study also confirmed that similar as AP-III-a4, treatment of the cancer cells with GRN A also leads to enhancement of glucose uptake. Further study reveals that the GRN A inhibited gluconeogenesis is attributed to the inhibition of PCK1 and PCK2, and increased expression of ENO1 reverses the effect of GRN A on the expression of PCK1 and PCK2. We also found that GRN A is able to inhibit the enzymatic activity of ENO1; treatment of the ENO1 solution with 0.1 μM GRN A results in 40% decrease of the enzymatic activity of ENO1 (data not shown). It is well known that gluconeogenesis plays an important role in maintaining blood glucose level, and the metabolism of glycogen as well as fatty acid. This study provides primary evidence that GRN A possesses potential to be developed as a novel regulator of blood glucose level. Since the level of ENO1 is correlated with the expression of PCK1 and PCK2, targeting ENO1 may be a novel strategy for regulating the glucose metabolism. However, the detailed function of GRN A in gluconeogenesis needed to be further addressed.

Metastasis, a characteristic unique of cancer cells, includes multiple discrete steps; local migration, intravasation, circulating to the target organ, extravasation into target organ site, multiplication in target organ [15, 16]. Migration and invasion of cancer cells result in the failure of standard chemotherapy and appearing to be inaccessible for irradiation or surgical treatment. Therefore, preventing invasion and metastasis of cancer cells is a promising strategy to develop novel anticancer agents. However, although a lot of antimetastasis agents have been found, no effective drugs are used clinically with good results [17]. Our present study showed that GRN A displays significant inhibition effects on migration and invasion of cancer cells. Since peptides from human cells display little immunogenicity, finding anticancer agents from human source is promising. Several peptides from human sources have been successfully used clinically for the treatment of human cancer, such as interferons and interleukins [18–20]. However, these kinds of peptides usually do not inhibit cancer cell growth directly; they affect the growth of tumors via an immune effect. In the present study, we found that the GRN A possesses the ability to inhibit proliferation of cancer cells directly. Furthermore, the peptide is also able to inhibit migration and invasion. This result provides primary evidence that GRN A has the potential to be developed as a novel kind of anticancer agent. In this study, we also confirmed that the inhibition effect of GRN A on cell migration and invasion depends on the interaction of GRN A and ENO1. This study also provides evidence that targeting ENO1 may be an important strategy to develop antimetastasis agents. Previous study also showed that ENO1 also functions as a plasminogen receptor; ENO1 can bind with plasminogen on the cell surface in a diverse of cells [9]. ENO1 was able to interact with uPA, and uPAR; resulting in augmentation of cell migration and invasion. More studies are needed to document if GRN A treatment of cancer cells is able to affect the interaction of ENO1 with plasminogen, uPA and uPAR.

Accumulative evidence reveals that in addition to its innate glycolytic functions, ENO1 plays important role in several biological and pathophysiological process; functioning as plasminogen receptor, playing role in myogenesis and muscle regeneration as well as in cell apoptosis etc [21], Higher expression of ENO1 is correlated with several kinds of cancer development [11, 12, 14, 22, 23]. However, the regulation of ENO1 is still not clear. Our study for the first time reveals that GRN A is able to interact with ENO1 and the interaction affects cell migration and invasion. The study reveals a novel regulation mechanism of ENO1; the function of ENO1 is regulated in part by GRN A. Studies are needed to address if the interaction of GRN A and ENO1 affects the other functions of ENO1.

## MATERIALS AND METHODS

### Cell culture

The cell lines, including human lung carcinoma cells NCI-H460 and A549, human pancreatic cancer cells PANC-28, MIA PaCa-2, human cervical cancer Hela cells, human hepatoma cells BEL-7402 and HepG-2, human melanoma cells A875, human epidermoid carcinoma cells A431 were obtained from ATCC and were cultured in Dulbecco's modified eagle's medium (DMEM, Corning, NY) supplemented with 10% fetal bovine serum (FBS, Corning, NY), with 5% CO_2_ at 37°C.

### Antibodies

Monoclonal antibodies; including rabbit anti-PCK1 (#12940), rabbit anti-PCK2 (#8586), rabbit anti-c-Myc (#13987) and the polyclonal rabbit anti-PARP (#9542) were purchased from Cell Signaling Technology (Danvers, MA). The monoclonal mouse anti-His-tag (#ab18184), anti-β-actin (#ab8229), anti-G-6-Pase (#ab83690) and anti-ENO1 (#ab112994) were purchased from Abcam (Cambridge, UK). The polyclonal rabbit anti-Bcl-xL (#AB126) and the polyclonal rabbit anti-rabbit AKT (#AA326) were obtained from Beyotime (Shanghai, China). The monoclonal mouse anti-β-tubulin (#CW0098M) and fluorescein (FITC)-conjugated affinipure goat anti-mouse IgG antibody (#121217) were products of ComWin Biotech (Beijing, China) and Jackson ImmunoResearch Laboratories (West Grove, PA) respectively.

### Cell cytotoxicity evaluation

MTT assay was performed to evaluate the anti-proliferative effects of GRN A against NCI-H460, A549, PANC-28, MIA PaCa-2, Hela, BEL-7402, HepG-2, A875 and A431 cells. Briefly, cells (5 × 10^3^) were seeded in 96-well culture plates and incubated at 37°C in humidified air atmosphere with 5% CO_2_. After incubation for 24 h, the cells were treated without or with certain concentrations of GRN A in serum-free DMEM media. After cultured for another 48 h or 72 h, MTT (10 μL, 5 mg/mL, Sigma-Aldrich, St. Louis, MO) was added to each well and cells were incubated for an additional 4 h. DMSO (150 μL) was added to each well to dissolve the reduced MTT crystals. The MTT-formazan product dissolved in DMSO was estimated by measuring the absorbance at 570 nm with a micro plate reader (Biotech, power wave, USA). The percentage of cell growth inhibition was calculated as follows:

Relative inhibitory rate (%) = (OD_control_–OD_treated_)/OD_control_ × 100%.

### Flow cytometry analysis

Flow cytometry analysis was performed using Annexin V-FITC/PI apoptosis detection kit (#KGA105, KeyGen, Nanjing, China) according to the manufacturer's protocol. Briefly, cells (2 × 10^5^) were seeded in 6-well culture plates and incubated for 24 h at 37°C. GRN A with certain concentration was directly added and incubated for an additional 48 h. Then cells were harvested and resuspended in phosphate buffered saline (PBS). Apoptotic cells were examined by Epics-XL/MCL flow cytometer (Beckman coulter, USA).

### Confocal imaging

HepG-2 cells (4 × 10^4^) were cultured on a 35 mm confocal dish (Corning, NY) and incubated at 37°C. After incubation for 24 h, the cells were treated without or with certain concentrations of GRN A. After cultured for another 24 h, cells were fixed with 4% paraformaldehyde and permeabilized with or without 0.1% triton X-100. The cells were further incubated with a mouse monoclonal anti-His-tag antibody overnight at 4°C. Subsequently, the cells were incubated with FITC-conjugated goat anti-mouse IgG antibody. After incubation for additional 4 h, 6-diamidino-2-phenylindole (DAPI) was added, and the cells were observed using a Leica TCS SP8 confocal system (Leica microsystems, Germany).

### Pull down assay

Pull-down experiment was performed using the Pull-down polyHis protein: protein Interaction kit (#21277, Pierce, IL) as per the manufacture's instruction. Briefly, HepG-2 cells (2 × 10^5^) were cultured on a 60 mm culture dish and incubated at 37°C for 24 h. The cells were released by trypsin digestion and washed with Tris-HCL buffered saline (TBS) and collected by centrifuging at 500 × g for 5 min. The cells were resuspended in 2.5 mL of ice-cold TBS per gram wet weight of cells using a pipette. ProFound^TM^ lysis buffer was added and mixed thoroughly on ice for 30 min and the lysate was collected by centrifugation at 12000 × g for 5 min. Imidazole stock solution (4 M) was added to adjust imidazole to a final concentration of 20 mM. Cobalt chelate resin (50 μL) was added into the spin column and equilibrated with washing solutions. Subsequently, the resin was incubated with or without 300 μL of His-GRN A (100 μg) at 4°C with gentle rocking. After incubated for 1 h, the resins were collected by centrifugation at 1200 × g for 1 min and washed 5 times with TBS and incubated with the crude lysates of HepG-2 cells for 4 h at 4°C. After washed with TBS for additional 5 times, the bound proteins in the resin were eluted with elution buffer (imidazole, 290 mM). The eluted sample was resolved using SDS-PAGE and visualized with Coomassie brilliant blue.

### Liquid chromatography-tandem mass spectrometry (LC-MS/MS) analysis

The protein band stained with Coomassie blue was excised from the polyacrylamide gel and destained with 40% acetonitrile/50 mM NH_4_HCO_3_. The gel pieces were dehydrated with 100% acetonitrile and reduced disulfide bonds with DTT (10 mM, 56°C, 45 min), and the free sulfhydryl groups were alkylated with iodoacetamide (55 mM, 25°C, 60 min in the dark). The band was digested using enzyme solution (100 ng/μL of trypsin in 25 mM NH_4_HCO_3_, pH 8.3) at 37°C overnight, and quenched with 60% acetonitrile containing 0.1% FA and concentrated to 25 μL using a speed vac.

The resulting tryptic peptides were reconstituted in HPLC using buffer A (0.5% formic acid water solution), loaded across a trap column (ReproSil-Pur C18-AQ, 0.15 × 30 mm, Dr. Maisch GmbH, Germany) at a flow rate of 0.2 μL/min and separated on a resolving analytical C18 column (ReproSil-Pur C18-AQ, 0.75 × 150 mm) with a linear gradient of buffer B from 4–8 0% (0.5% formic acid acetonitrile) at a flow rate of 300 nL/min. Eluted peptides were analyzed with nanoLC-LTQ-Orbitrap XL mass spectrometers (Thermo, San Jose, CA), and detected in the Orbitrap at a resolution of 60,000. Eluted peptide cations were converted to gas-phase ions by Nanospray Flex ion source with 2.1 kV, and survey full scan MS spectra were acquired from *m/z* 300 to *m/z* 1800. The raw data was processed using Proteome Discoverer (version 1.4.0.288, Thermo Fischer Scientific).

### Western blotting assay

Samples from pull-down assay were resolved in a 12% polyacrylamide gel and then transferred onto a PVDF membrane (Millipore, MA). After being blocked with 5% nonfat milk in TBS-T buffer (20 mM Tris, 137 mM sodium chloride at pH 7.6, 0.1% Tween-20) for 1 h at room temperature, membranes were incubated with primary antibody overnight at 4°C. Horse radish peroxidase (HRP)-conjugated (ZSGBBIO, Beijing, China) secondary antibodies were used to detect the immunoreactivity by enhanced chemiluminescence (ECL) detection reagents (Applygen Technologies, Beijing, China).

Western blotting analysis was also performed to determine the expression of certain apoptotic related or glucose-metabolism related proteins in cells treated with or without GRN A. Briefly, HepG-2 cells were seeded into 6 well plates at a density of 2 × 10^5^ cell/well. After treated with or without GRN A for 48 h, the cells were collected and lysed with cold RIPA buffer (#R0278, Sigma-Aldrich, St. Louis, MO). The cell lysates were resolved on SDS-PAGE and analyzed using Western blotting experiments.

### SPR interaction analysis

ENO1 (#ATGP0404, ATGen, Montevideo, Uruguay) was diluted in 10 mM sodium acetate buffer at PH 5.0 and 8171 response units (RU) were directly immobilized on a CM5 biosensor chip (Biacore AB, Uppsala, Sweden). GRN A was sequentially diluted in HBS-EP buffer (10 mM HEPES, 150 mM NaCl, 3 mM EDTA and 0.05% (v/v) surfactant P20) and used as a mobile phase and injected over the protein surface with certain concentrations of GRN A (3.5, 7.0, 14.0, 28.0, 56.0, 112.0 μM) at rate of 30 μL/min. The flow cell without immobilized protein was served as a non-specific binding control. Binding was monitored using a BIACORE T200 (GE Healthcare, Uppsala, Sweden). The parameters K_D_ (equilibrium dissociation constant) was determined by the Biacore T200 evaluation software version 2.0 using the formulae: K_D_ = K_off_/K_on_ (K_on_ = association rate constant and K_off_= dissociation rate constant)

### Measurement of the level of glucose uptake

Intracellular glucose uptake levels were determined using a glucose assays kit (#DIGL-100, BioAssay Systems, CA), according to the manufacturer's protocol. Briefly, HepG-2 cells (1 × 10^5^) were cultured on a 60 mm culture dish and incubated at 37°C for 24 h, and then the cells were treated with or without GRN A (5 μM) in 1% serum DMEM. After incubation for 0, 6, 12, 24, 48 and 72 h, the cells were harvested and washed with PBS, and then the cells were lysed with RIPA buffer and collected by centrifugation (10000 × g for 20 min). Total protein concentration in the supernatant was determined using BCA protein assay kit (#P0012s, Beyotime, China). To determine the levels of glucose uptake, the culture media (5 μL) were added into 500 μL working solution. After heating for 8 min, the sample was cooled down in cold water bath for 4 min, the mixture samples (200 μL) were transferred into a 96-well plate and the glucose uptake was determined by measuring the absorbance at 630 nm with a micro plate reader (Biotech, power wave, USA).

### Scratch wound healing assay

HepG-2 cells were seeded in a 6-well plate. After grown to 100% confluence, wounds were made by scraping the monolayer of cells with a 200 μL tip. GRN A with certain concentration was added and cultured for 0, 6, 12, and 24 h. The area between wound edges in each well was measured using a standard template placed on the image. The wounded area was determined using Image Pro Plus Software.

### Cancer cell invasion and migration assay

Transwell assay was performed as described previously [24]. Briefly, HepG-2 cells (2 × 10^4^) were seeded into the upper chamber of a 24-well Transwell chamber (#3422, Corning, NY,). An aliquot of 800 μL culture medium supplemented with 10% FBS was added into the lower well of the chamber. After incubation for 4 h, the cells were treated without or with certain concentrations of GRN A in DMEM media containing 1% FBS. After incubation for 48 h, cells in the upper well were removed with a cotton swab. Cells that migrated into the lower well were washed with PBS, fixed in 4% paraformaldehyde and stained with 0.25% crystal violet. Cell migration was quantified by counting the migrated cells in microscopic fields (100X) per filter, and the mean value per filter was calculated from four replicate filters.

For Transwell invasion assay, the cells of the upper well of the transwell were coated with 50 μL (1 μg/mL) Matrigel (#356234, BD, CA). The Matrigel was allowed to harden at 37°C in a 5% CO_2_ incubator for 4 h and then HepG-2 cells (2 × 10^4^) were seeded into the upper chamber of a 24-well Transwell chamber. The rest of the assay was performed as described above.

### Statistical analysis

Statistical analyses were performed using the software Excel 2007 and SPSS 20.0. Results are expressed as mean ± SD. One-way analysis of variance followed by Tukey's multiple comparison test was used to determine statistical significances. Statistical significance was accepted for *p < 0.05.
